# Agent-Specific Clinical Profiles of Antibiotic-Associated Encephalopathy: A Dual-Database Pharmacovigilance Study Using JADER and FAERS

**DOI:** 10.3390/antibiotics15090917

**Published:** 2026-09-17

**Authors:** Hideya Itagaki

**Affiliations:** Division of Emergency and Disaster Medicine, Tohoku Medical and Pharmaceutical University Hospital, 1-15-1 Fukumuro, Miyagino-ku, Sendai 983-8512, Miyagi, Japan; hideya.itagaki@gmail.com; Tel.: +81-22-259-1221

**Keywords:** antibiotic-associated encephalopathy, pharmacovigilance, JADER, FAERS, reporting odds ratio, metronidazole, cefepime, disproportionality analysis

## Abstract

**Background/Objectives:** Antibiotic-associated encephalopathy (AAE) is a serious adverse reaction whose presentation is thought to differ between antibiotics; whether these agent-specific profiles are consistently reflected in spontaneous reports across independent data sources is unknown. **Methods:** Using the Japanese Adverse Drug Event Report (JADER) database and the US FDA Adverse Event Reporting System (FAERS, 2004–2026), we studied metronidazole, cefepime, ceftriaxone, ceftazidime, and levofloxacin. Encephalopathy was defined by the MedDRA terms Encephalopathy or Toxic encephalopathy, and reporting odds ratios (RORs) were calculated in each database; co-reported neurological manifestations, time to onset, patient age, and renal-associated terms were then characterized. **Results:** All five antibiotics showed significant reporting disproportionality in both databases, with metronidazole and cefepime showing the largest RORs in each (JADER: metronidazole ROR 186.70, cefepime 55.33; FAERS: cefepime 69.29, metronidazole 29.11), consistent with established adverse reactions. In FAERS, metronidazole showed a cerebellar profile (ataxia OR 12.48, dysarthria OR 11.11) and cefepime a renal-associated altered-mental-status/myoclonus profile (depressed level of consciousness OR 12.88, myoclonus OR 11.76; renal-associated terms OR 2.88; median age 71 years); event-specific JADER dates indicated a delayed course for metronidazole (median 28 days). The symptom-level contrast was FAERS-based and was not reproduced when the same analysis was repeated in JADER, a discrepancy that may reflect differences in coding practice. **Conclusions:** AAE is reported not as a single uniform event but with profiles specific to the causative antibiotic. Awareness of these agent-specific patterns may support earlier recognition, although confirmation in analytic studies with exposure denominators is warranted.

## 1. Introduction

The neurotoxic potential of antibiotics has been recognized since the earliest years of their clinical use. Psychiatric disturbances were reported with systemic penicillin as early as 1948 [1], and by the 1960s high-dose intravenous penicillin had become established as a cause of “penicillin encephalopathy,” characterized by myoclonus, hyperreflexia, seizures, and coma [2]. Subsequently, neurological and psychiatric adverse events were described with quinolones and metronidazole [3,4]. Building on these observations, Bhattacharyya et al., in a comprehensive 2016 review, proposed a clinical classification of antibiotic-associated encephalopathy (AAE) comprising three phenotypes: (i) an early-onset seizure/myoclonus type associated with penicillins and cephalosporins; (ii) an early psychiatric type associated with quinolones, sulfonamides, and macrolides; and (iii) a delayed cerebellar type with characteristic magnetic resonance imaging (MRI) abnormalities attributed to metronidazole [5].

AAE is clinically important but easily missed, because its features overlap with those of sepsis, metabolic derangement, and the underlying infection; delayed recognition may prolong hospitalization and contribute to preventable morbidity and mortality [5,6]. Beyond their differing clinical pictures, these syndromes also diverge in mechanism and risk factors—cefepime neurotoxicity is driven by GABA-A receptor antagonism and is closely tied to impaired renal clearance [7], whereas metronidazole toxicity is dose- and duration-dependent [8]—reinforcing the view that AAE differs systematically between antibiotics.

Spontaneous reporting systems, such as the Japanese Adverse Drug Event Report (JADER) database and the US FDA Adverse Event Reporting System (FAERS), accumulate large numbers of reports of rare reactions such as AAE and therefore allow their reported clinical features to be characterized at scale, although they lack exposure denominators and cannot estimate incidence or risk. Prior pharmacovigilance work on AAE has nevertheless been limited. Studies have generally used a single database, and the most comprehensive to date—a FAERS-based screen of 121 antibiotics—assigned drugs to the three types from prior literature rather than deriving their reported profiles from the data [9]. Whether the agent-specific presentations of AAE described in the clinical literature are consistently reflected in spontaneous reports, and whether they are reproducible across independent reporting systems, therefore remains unexamined. Notably, the neurotoxicity of cefepime and metronidazole is already an established adverse reaction; the question is therefore not whether it exists but how the reported clinical picture differs between agents.

We therefore conducted a dual-database study using JADER and FAERS on five clinically important antibiotics with recognized or suspected neurotoxicity—metronidazole, cefepime, ceftriaxone, ceftazidime, and levofloxacin. The study had two aims: first, to assess whether reporting disproportionality for encephalopathy is consistently observed in both systems; and second, to characterize and compare the reported clinical profile of encephalopathy for each agent (co-reported neurological manifestations, time to onset, patient age, renal-associated factors, laboratory-abnormality-related terms, and seriousness criteria of the reports), and thereby to examine whether AAE is reported as a single uniform adverse event or with agent-specific profiles.

## 2. Materials and Methods

### 2.1. Study Design and Data Sources

**Figure 1:**
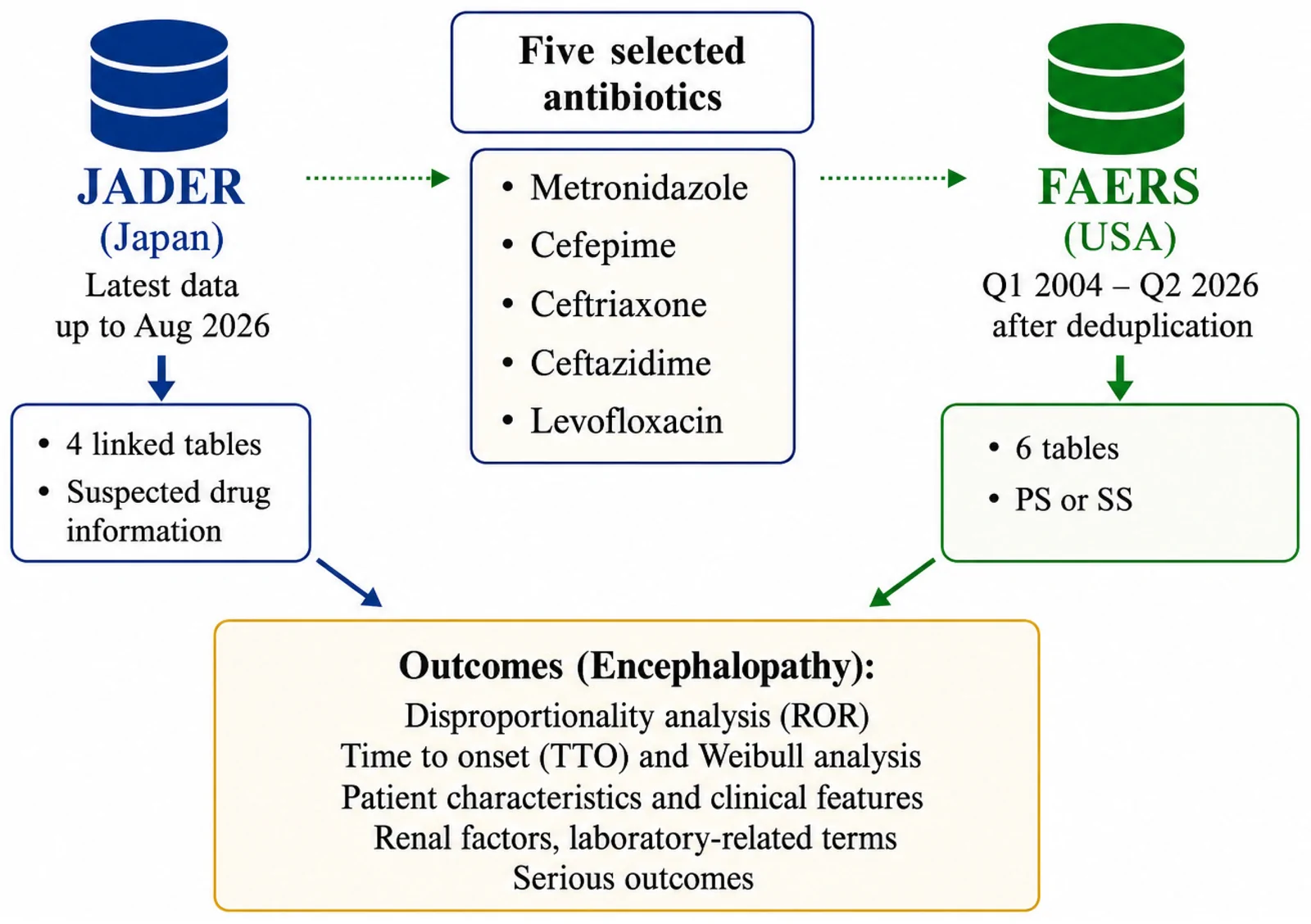
Overview of the study design and data sources. Dotted arrows indicate the antibiotics selected for analysis; solid arrows indicate the flow of data. Five antibiotics were analyzed in two independent spontaneous reporting systems, JADER (Japan) and FAERS (USA), for encephalopathy across disproportionality, time-to-onset and Weibull, patient characteristics, renal and laboratory factors, and serious outcomes.

We conducted a retrospective pharmacovigilance study using two independent spontaneous reporting systems: the Japanese Adverse Drug Event Report (JADER) database and the US Food and Drug Administration Adverse Event Reporting System (FAERS). JADER is a publicly available spontaneous adverse drug reaction database maintained by the Pharmaceuticals and Medical Devices Agency (PMDA) of Japan. All reports available in the JADER dataset downloaded in August 2026 were used. The database consists of four linked tables containing demographic information, drug information, adverse events, and underlying diseases. For FAERS, quarterly ASCII data files from the first quarter of 2004 through the second quarter of 2026 were obtained. The DEMO, DRUG, REAC, THER, INDI, and OUTC tables were used for demographic characteristics, drug exposure, adverse events, treatment dates, indications, and clinical outcomes, respectively. Both databases consist of anonymized, publicly available spontaneous reports; therefore, institutional review board approval and informed consent were not required. The study was designed and reported in accordance with the Reporting of a Disproportionality Analysis for Drug Safety Signal Detection Using Individual Case Safety Reports in Pharmacovigilance (READUS-PV) recommendations. An overview of the study design and data sources is shown in Figure 1.

### 2.2. Data Cleaning and Deduplication

For JADER, records were linked across the demographic, drug, adverse-event, and underlying-disease tables using the case identification number, and analyses were performed at the unique case level. FAERS reports were deduplicated before analysis. For each CASEID, the report with the most recent FDA_DT was retained. When multiple reports for the same CASEID had the same FDA_DT, the report with the largest PRIMARYID or, for older AERS records, ISR was retained. Cases included in FDA deleted-case files were subsequently excluded.

### 2.3. Antibiotic Exposure

Five antibiotics were selected for detailed analysis: metronidazole, cefepime, ceftriaxone, ceftazidime, and levofloxacin. These agents were selected because they represent clinically relevant antibiotics with recognized or suspected neurotoxicity and had sufficient numbers of encephalopathy reports to permit comparative analyses in both databases. This selection was pragmatic and not intended to be exhaustive: other antibiotics for which neurotoxicity has been reported—including other carbapenems, penicillins, and fluoroquinolones—were not examined, and the findings should not be generalized beyond the five agents studied. In JADER, exposure was defined as the antibiotic being classified as a suspected drug. In FAERS, exposure was defined as the antibiotic being designated as either a primary suspect (PS) or secondary suspect (SS) drug. Generic names and major trade names were harmonized to the corresponding active ingredient.

### 2.4. Definition of Encephalopathy

The primary outcome was antibiotic-associated encephalopathy, defined by the MedDRA Preferred Terms (PTs) Encephalopathy or Toxic encephalopathy. A report containing either PT was classified as an encephalopathy report. Because the PT Toxic encephalopathy may be preferentially used for adverse events considered drug-related and may therefore contribute to terminology-related reporting bias, a sensitivity analysis was conducted using Encephalopathy alone as the outcome.

### 2.5. Disproportionality Analysis

Reporting disproportionality was assessed using the reporting odds ratio (ROR) and its 95% confidence interval (CI). For each antibiotic, a conventional 2 × 2 contingency table was constructed based on the presence or absence of antibiotic exposure and encephalopathy. Reporting disproportionality was considered significant when the lower limit of the 95% CI of the ROR exceeded 1. RORs were interpreted as measures of reporting disproportionality rather than estimates of incidence, relative risk, or causal association. Because spontaneous reporting systems provide no exposure denominators and RORs are influenced by prescribing frequency, indication, treatment duration, healthcare setting, and reporting practices, RORs were not compared between antibiotics as estimates of the relative magnitude of risk [10]. Analyses were performed separately in JADER and FAERS to evaluate the consistency of reporting disproportionality across two independent spontaneous reporting systems.

### 2.6. Time-to-Onset Analysis

Time to onset (TTO) was evaluated among reports for which sufficiently precise treatment initiation and event-onset dates were available. In JADER, TTO was calculated as the interval between the recorded antibiotic treatment start date and the onset date of encephalopathy. In FAERS, TTO was calculated as the number of days from the treatment start date recorded in the THER table (START_DT) to the event date recorded in the DEMO table (EVENT_DT); only reports with exact calendar dates in YYYYMMDD format were included, and reports with an event date preceding the treatment start date (TTO < 0) were excluded. TTO was summarized using the median and interquartile range (IQR), and the proportions of reports with an event date occurring within 7, 14, and 30 days after treatment initiation were calculated. Temporal patterns were further evaluated using a two-parameter Weibull distribution; the shape parameter (β) and its 95% CI were estimated by maximum likelihood, with profile-likelihood CIs. A 95% CI entirely below 1 was interpreted as an early-failure pattern, one including 1 as a random-failure pattern, and one entirely above 1 as a wear-out pattern. Because EVENT_DT in FAERS represents a report-level event date and does not necessarily correspond to the onset date of the encephalopathy PT itself, the FAERS TTO and Weibull analyses were considered exploratory.

### 2.7. Patient Characteristics and Renal-Associated Factors

Age and sex were summarized among encephalopathy reports when available. In FAERS, renal-associated factors were identified using co-reported MedDRA terms related to renal impairment, chronic kidney disease, acute kidney injury, renal failure, dialysis, and hemodialysis. Within each antibiotic-specific FAERS cohort, the frequency of these renal-associated terms was compared between reports with and without encephalopathy, and crude odds ratios with 95% CIs were calculated. Because these terms were derived from spontaneously reported adverse-event information, they were interpreted as co-reported renal-associated factors rather than systematically ascertained baseline comorbidities.

### 2.8. Laboratory-Abnormality-Related Terms

FAERS does not provide structured numerical laboratory measurements such as serum creatinine or estimated glomerular filtration rate. We therefore explored co-reported MedDRA PTs reflecting laboratory abnormalities. Prespecified abnormalities included terms related to increased blood creatinine, decreased glomerular filtration rate, increased blood urea, hyponatremia or decreased blood sodium, hypernatremia, hypokalemia, hyperkalemia, hypoglycemia, hyperglycemia, increased hepatic enzymes, increased alanine aminotransferase, increased aspartate aminotransferase, and increased bilirubin. These analyses were considered exploratory and were not interpreted as analyses of actual laboratory values.

### 2.9. Co-Reported Neurological Manifestations

To characterize the reported clinical profile of AAE, prespecified co-reported neurological manifestations were examined, including ataxia, dysarthria, gait disturbance, myoclonus, confusional state, depressed level of consciousness, delirium, and seizure. For FAERS, the proportion of each manifestation was compared between encephalopathy and non-encephalopathy reports within the same antibiotic cohort, and crude ORs with 95% CIs were calculated. Because spontaneous reporting databases do not consistently provide a reliable chronological sequence for individual PTs, these manifestations were considered co-reported neurological manifestations and not necessarily presenting symptoms preceding the diagnosis of encephalopathy. To assess whether this symptom-level contrast was reproduced in Japanese data, the same within-drug comparison was repeated in JADER for metronidazole and cefepime as a sensitivity analysis, comparing the frequency of the prespecified manifestations between encephalopathy and non-encephalopathy reports of each antibiotic (Supplementary Table S5). This analysis compared the frequency of prespecified manifestations between groups and did not statistically identify phenotypes by clustering or similar methods.

### 2.10. Seriousness Criteria of Reports

Seriousness criteria of the reports were assessed using FAERS OUTC records. The prespecified criteria were death, hospitalization, and life-threatening events. Individual reports could contain more than one criterion; therefore, these categories were not mutually exclusive. These criteria indicate the seriousness of the case in which encephalopathy was reported; the severity of the encephalopathy itself is not recorded in FAERS.

### 2.11. Statistical Analysis

Categorical variables were summarized as frequencies and percentages and continuous variables as medians with IQRs, as appropriate. RORs and crude within-antibiotic ORs were reported with 95% CIs. Weibull parameters were estimated using maximum likelihood methods, with profile-likelihood CIs for the shape parameter. Because analyses of renal factors, laboratory-abnormality-related terms, and individual neurological manifestations were exploratory and involved multiple comparisons, these findings were interpreted primarily according to effect size, precision, biological plausibility, and consistency across analyses rather than statistical significance alone.

### 2.12. Data Processing and Reproducibility

All data processing and analyses were performed in Python version 3.13.5 (Python Software Foundation, Beaverton, OR, USA) with pandas version 2.2.3 and NumPy version 2.3.5 (NumFOCUS, Austin, TX, USA). The FAERS case-level deduplication, the disproportionality (ROR) analyses, the within-drug odds ratios for co-reported manifestations, and the time-to-onset and Weibull analyses were implemented following the prespecified term definitions and the procedures described in this section and in Section 2.1, Section 2.2, Section 2.3, Section 2.4, Section 2.5, Section 2.6, Section 2.7, Section 2.8, Section 2.9, Section 2.10 and Section 2.11. The analysis code is available from the author on reasonable request.

## 3. Results

### 3.1. Reporting Disproportionality in JADER

**Figure 2:**
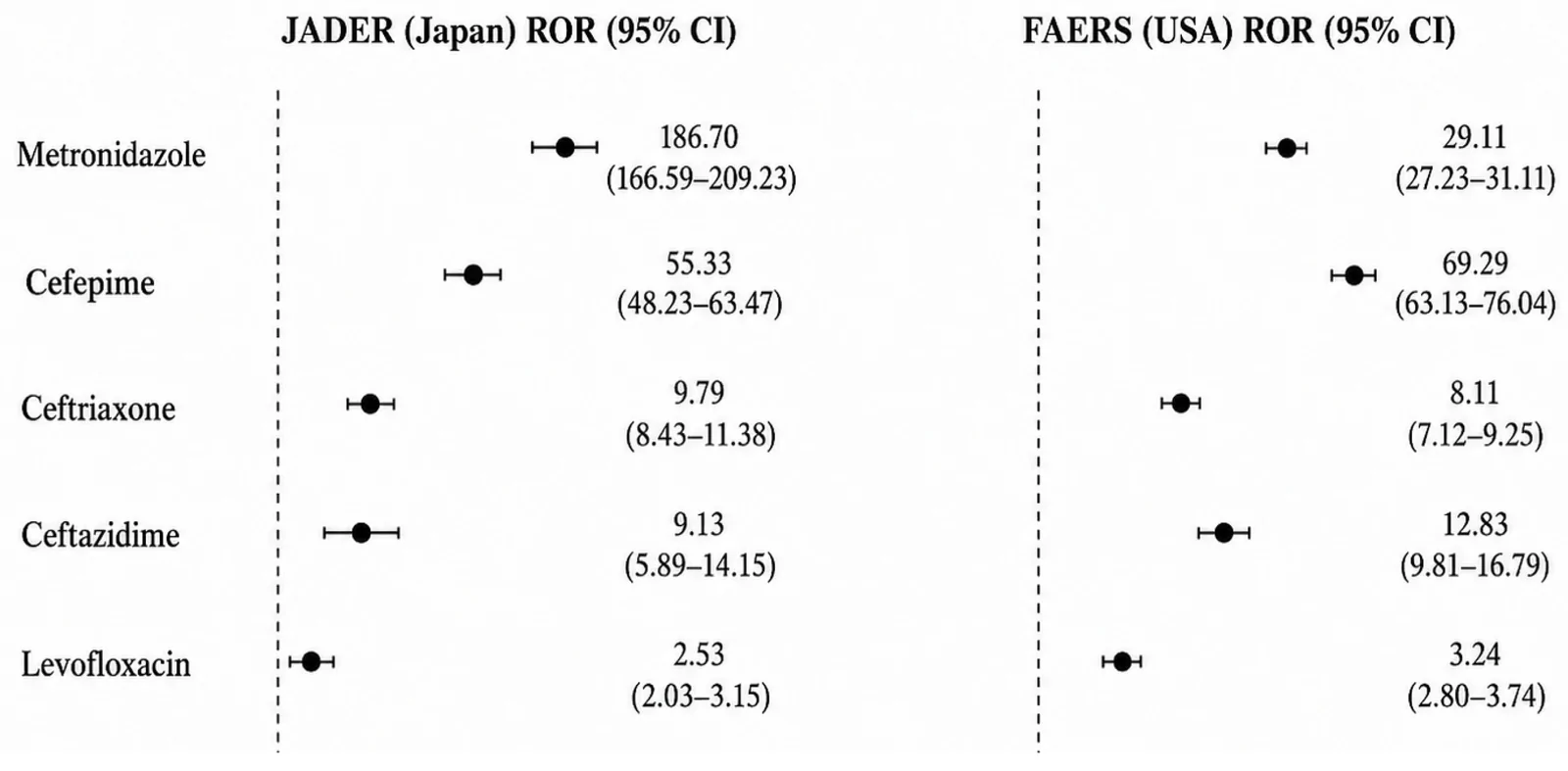
Reporting odds ratios (RORs) with 95% confidence intervals for encephalopathy associated with the five antibiotics in JADER and FAERS. All five antibiotics showed significant reporting disproportionality in both databases.

In JADER, significant reporting disproportionality for encephalopathy was observed for all five antibiotics analyzed. Metronidazole showed the largest ROR, with 586 encephalopathy reports among 1269 suspected-drug reports, corresponding to an ROR of 186.70 (95% CI 166.59–209.23). Cefepime also showed marked disproportionality, with 270 encephalopathy reports among 1266 suspected-drug reports and an ROR of 55.33 (95% CI 48.23–63.47). Significant reporting disproportionality was also observed for ceftriaxone (ROR 9.79, 95% CI 8.43–11.38), ceftazidime (ROR 9.13, 95% CI 5.89–14.15), and levofloxacin (ROR 2.53, 95% CI 2.03–3.15). Overall, all five selected antibiotics showed significant reporting disproportionality for encephalopathy in JADER, with particularly large RORs for metronidazole and cefepime (Table 1, Figure 2).

When the outcome definition was restricted to PT Encephalopathy alone, significant reporting disproportionality remained for all five antibiotics, although the magnitude of the RORs was attenuated. The RORs were 32.59 (95% CI 26.43–40.19) for metronidazole, 20.82 (95% CI 16.18–26.80) for cefepime, 4.88 (95% CI 2.18–10.94) for ceftazidime, 4.60 (95% CI 3.44–6.14) for ceftriaxone, and 1.71 (95% CI 1.19–2.47) for levofloxacin.

### 3.2. Time-to-Onset Patterns in JADER

Marked differences in time to onset (TTO) were observed between antibiotics (Table 2). For metronidazole, 145 reports contained evaluable TTO information; the median onset was 28 days (IQR 14–46), and only 13.1% occurred within 7 days, whereas 57.9% occurred within 30 days. In contrast, cefepime-associated encephalopathy occurred substantially earlier: among 78 evaluable cases, the median TTO was 4 days (IQR 3–6), with 80.8% occurring within 7 days, 94.9% within 14 days, and 98.7% within 30 days. Ceftriaxone similarly showed an early onset, with a median TTO of 5.5 days (IQR 4.25–7) among 38 evaluable reports and 84.2% occurring within 7 days. For ceftazidime, the median TTO was 3.5 days (IQR 2–6.25), although only four cases had evaluable dates. These findings indicated a comparatively delayed temporal pattern for metronidazole and an early-onset pattern for cephalosporin-associated encephalopathy.

### 3.3. Reporting Disproportionality in FAERS

After deduplication and exclusion of deleted reports, the FAERS analytic population comprised 20,435,080 unique cases, of which 23,015 contained either Encephalopathy or Toxic encephalopathy. Significant reporting disproportionality was observed for all five antibiotics. Cefepime showed the largest ROR, with 488 encephalopathy reports among 6868 PS/SS reports, corresponding to an ROR of 69.29 (95% CI 63.13–76.04). Metronidazole also showed marked disproportionality, with 933 encephalopathy reports among 30,522 PS/SS reports and an ROR of 29.11 (95% CI 27.23–31.11). Significant reporting disproportionality was also observed for ceftazidime (ROR 12.83, 95% CI 9.81–16.79), ceftriaxone (ROR 8.11, 95% CI 7.12–9.25), and levofloxacin (ROR 3.24, 95% CI 2.80–3.74). Overall, all five antibiotics showed significant reporting disproportionality for encephalopathy in FAERS, with particularly large RORs for cefepime and metronidazole (Table 1, Figure 2).

### 3.4. Cross-Database Consistency of Reporting Disproportionality

The magnitude of reporting disproportionality varied between JADER and FAERS, but significant disproportionality was observed for all five antibiotics in both databases. The RORs for ceftriaxone, ceftazidime, and levofloxacin were of broadly similar magnitude across the two databases. Metronidazole showed a substantially larger ROR in JADER, whereas cefepime showed the largest ROR in FAERS. Overall, the largest RORs in both databases were observed for metronidazole and cefepime (Table 1, Figure 2).

### 3.5. Patient Characteristics in FAERS

Patients with cephalosporin-associated encephalopathy tended to be older. The median age was 65 years (IQR 50–74) for metronidazole, 71 years (IQR 62–79) for cefepime, 68 years (IQR 56–78) for ceftriaxone, 77.5 years (IQR 63–81) for ceftazidime, and 63 years (IQR 54.3–75) for levofloxacin. Among cefepime-associated encephalopathy reports with available sex information, 195 were male, and 261 were female.

### 3.6. Co-Reported Neurological Manifestations in FAERS and JADER

**Figure 3:**
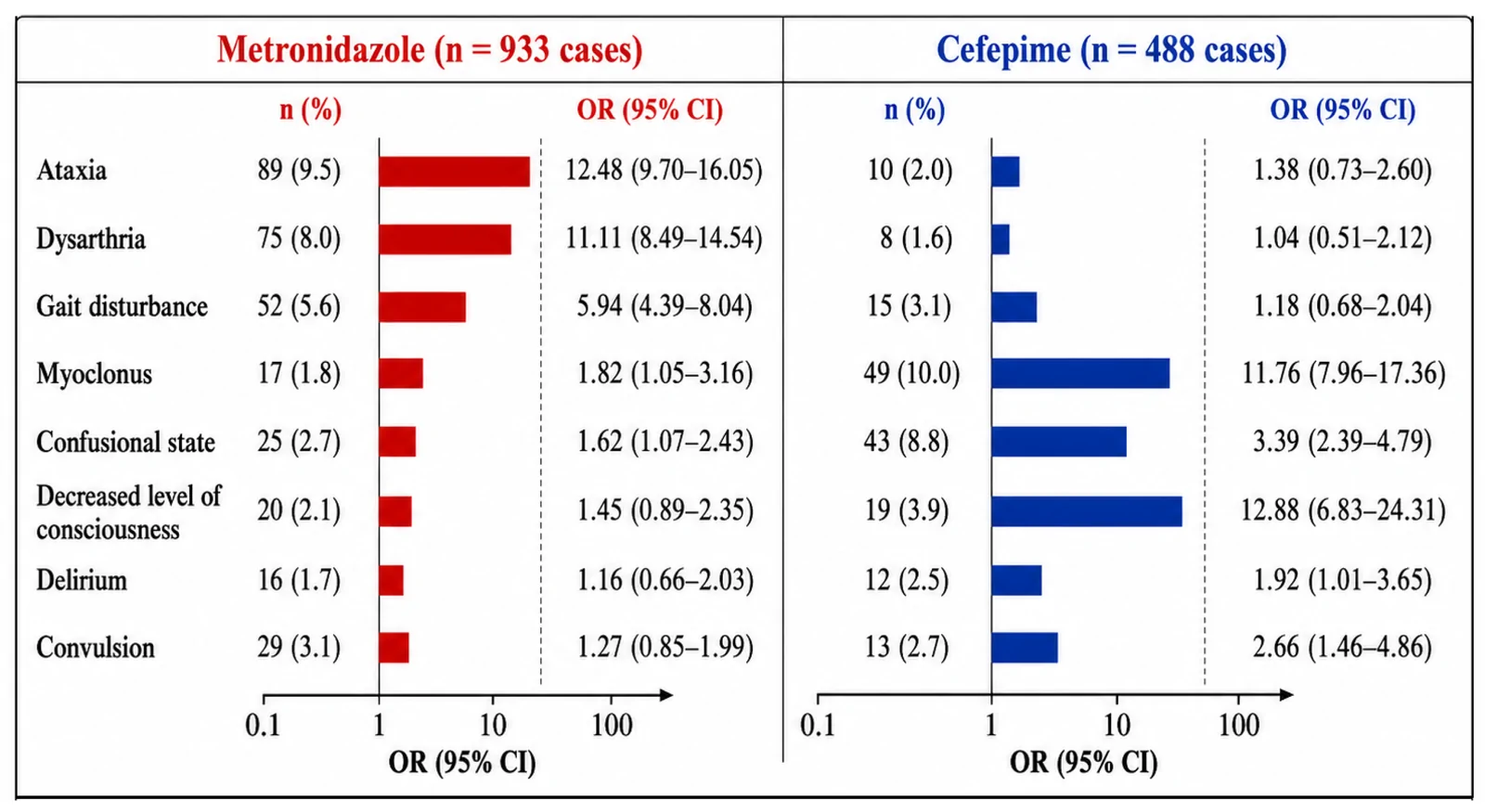
Co-reported neurological manifestations in metronidazole- versus cefepime-associated encephalopathy (FAERS). Cerebellar symptoms (ataxia, dysarthria, gait disturbance) predominated for metronidazole, whereas decreased consciousness and myoclonus predominated for cefepime. Odds ratios compare reports with versus without encephalopathy within each antibiotic.

Marked differences in co-reported neurological manifestations were observed between metronidazole and cefepime. Among 933 metronidazole-associated encephalopathy reports, ataxia was reported in 89 cases (9.5%), dysarthria in 75 (8.0%), gait disturbance in 52 (5.6%), seizure in 29 (3.1%), confusional state in 25 (2.7%), and myoclonus in 17 (1.8%). Compared with metronidazole reports without encephalopathy, particularly strong associations were observed for ataxia (OR 12.48, 95% CI 9.70–16.05), dysarthria (OR 11.11, 95% CI 8.49–14.54), and gait disturbance (OR 5.94, 95% CI 4.39–8.04). Among 488 cefepime-associated encephalopathy reports, myoclonus was reported in 49 cases (10.0%), confusional state in 43 (8.8%), depressed level of consciousness in 19 (3.9%), and seizure in 13 (2.7%). Compared with cefepime reports without encephalopathy, strong associations were observed for depressed level of consciousness (OR 12.88, 95% CI 6.83–24.31), myoclonus (OR 11.76, 95% CI 7.96–17.36), confusional state (OR 3.39, 95% CI 2.39–4.79), and seizure (OR 2.66, 95% CI 1.46–4.86). These patterns indicated distinct profiles of co-reported neurological manifestations for metronidazole and cefepime (Figure 3).

To examine whether this symptom-level contrast was also present in Japanese data, the same within-drug comparison was repeated in JADER (Supplementary Table S5). In JADER, however, the prespecified co-reported neurological Preferred Terms were uncommon for both agents (all <2%), and the FAERS symptom contrast was not reproduced. Among metronidazole-associated encephalopathy reports (*N* = 586), ataxia (0.7%), dysarthria (0.9%), and gait disturbance (0.9%) were each recorded in fewer than 1% of reports, and among cefepime-associated encephalopathy reports (*N* = 270), myoclonus was not recorded and depressed level of consciousness was recorded in 1.9% (5 reports). One possible explanation for this discrepancy is a difference in coding practice—in JADER, encephalopathy may be reported as a single term without enumerating the component neurological manifestations—although a true between-population difference in the reported phenotype cannot be excluded. The agent-specific symptom profiles are therefore presented as FAERS-based findings and not as findings reproduced across both databases.

### 3.7. Renal-Associated Factors in FAERS

**Figure 4:**
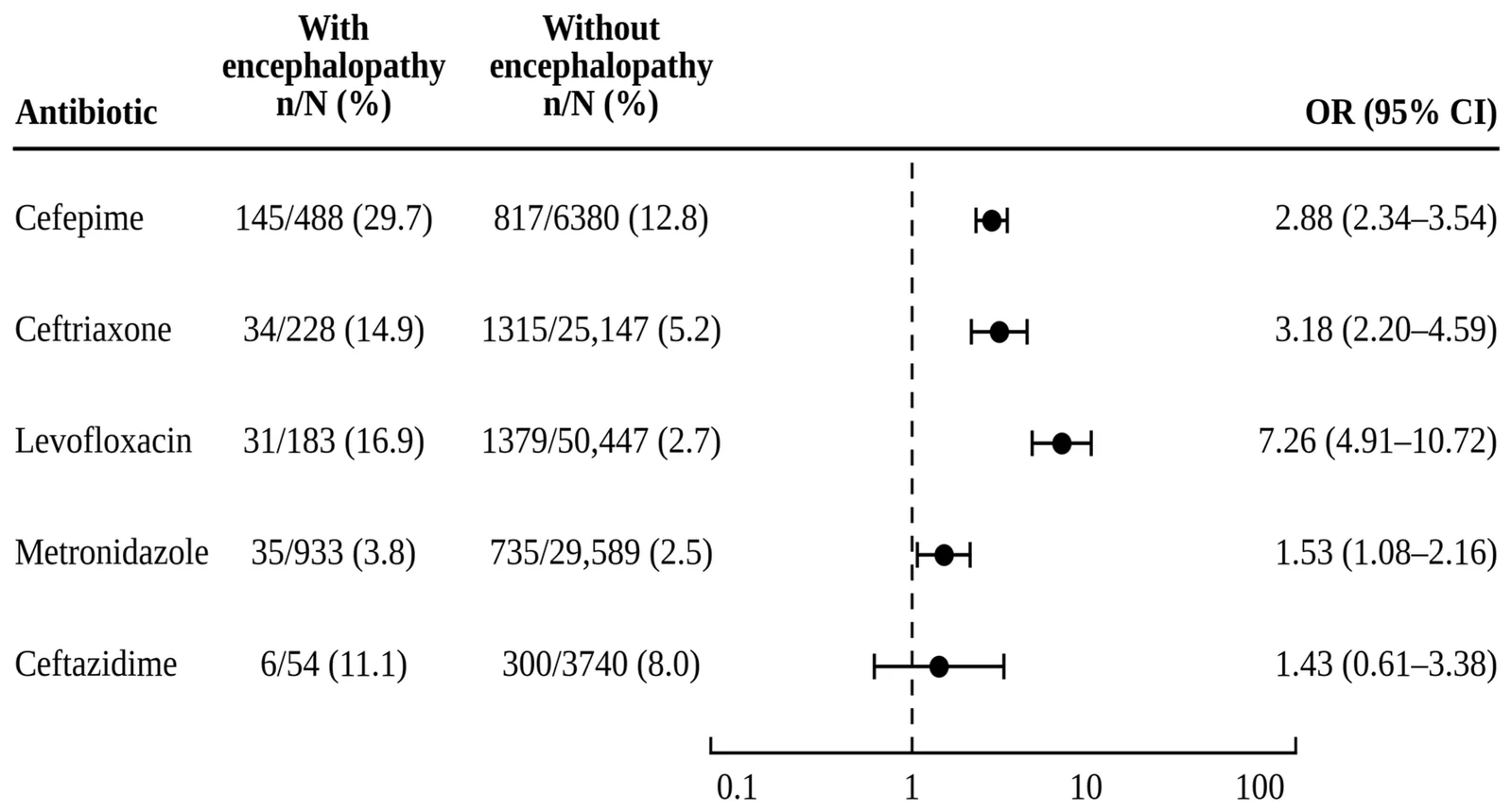
Odds ratios for co-reported renal-associated terms in encephalopathy versus non-encephalopathy reports, by antibiotic (FAERS). Renal-related terms were reported more frequently in encephalopathy cases for most antibiotics, especially cefepime and levofloxacin. The dashed vertical line indicates an odds ratio of 1 (no association). Renal-associated terms comprised renal impairment, chronic kidney disease, acute kidney injury, renal failure, dialysis, and hemodialysis.

Renal-associated terms were more frequently co-reported among encephalopathy reports for several antibiotics. For cefepime, renal-associated terms were reported in 145 of 488 encephalopathy reports (29.7%) compared with 817 of 6380 non-encephalopathy reports (12.8%), corresponding to an OR of 2.88 (95% CI 2.34–3.54). Dialysis-related terms were also more frequent among cefepime-associated encephalopathy reports, occurring in 7 of 488 reports (1.43%) compared with 34 of 6380 non-encephalopathy reports (0.53%), with an OR of 2.72 (95% CI 1.20–6.16). Renal-associated reporting was also elevated for ceftriaxone (OR 3.18, 95% CI 2.20–4.59), levofloxacin (OR 7.26, 95% CI 4.91–10.72), and metronidazole (OR 1.53, 95% CI 1.08–2.16). No clear association was observed for ceftazidime (OR 1.43, 95% CI 0.61–3.38) (Figure 4).

### 3.8. Laboratory-Abnormality-Related Terms in FAERS

Among cefepime reports, increased-creatinine-related terms were recorded in 9 of 488 encephalopathy reports (1.84%) and 62 of 6380 non-encephalopathy reports (0.97%), corresponding to an OR of 1.91 (95% CI 0.95–3.88). Decreased-GFR-related terms were uncommon but were more frequently reported among encephalopathy cases, occurring in 4 of 488 reports (0.82%) compared with 4 of 6380 non-encephalopathy reports (0.06%), with an OR of 13.17 (95% CI 3.28–52.84); because of the small number of reports, this finding was considered exploratory. Among levofloxacin reports, hyperkalemia-related terms were recorded in 9 of 183 encephalopathy reports (4.92%) and 62 of 50,447 non-encephalopathy reports (0.12%), corresponding to an OR of 42.03 (95% CI 20.57–85.91). Given the potential for confounding by renal dysfunction, disease severity, infection, and concomitant medications, this finding was interpreted cautiously.

### 3.9. Time-to-Onset Patterns in FAERS

Exact-date TTO was evaluable in 109 metronidazole reports, 265 cefepime reports, 61 ceftriaxone reports, 42 ceftazidime reports, and 77 levofloxacin reports. The median TTO was 8 days (IQR 4–21) for metronidazole and 6 days for cefepime (IQR 4–14), ceftriaxone (IQR 1–12), ceftazidime (IQR 4.25–12), and levofloxacin (IQR 4–13). Events were recorded within 30 days in 83.5% of metronidazole reports, 96.6% of cefepime reports, 93.4% of ceftriaxone reports, 95.2% of ceftazidime reports, and 97.4% of levofloxacin reports (Table 3).

### 3.10. Weibull Analysis in FAERS

**Figure 5:**
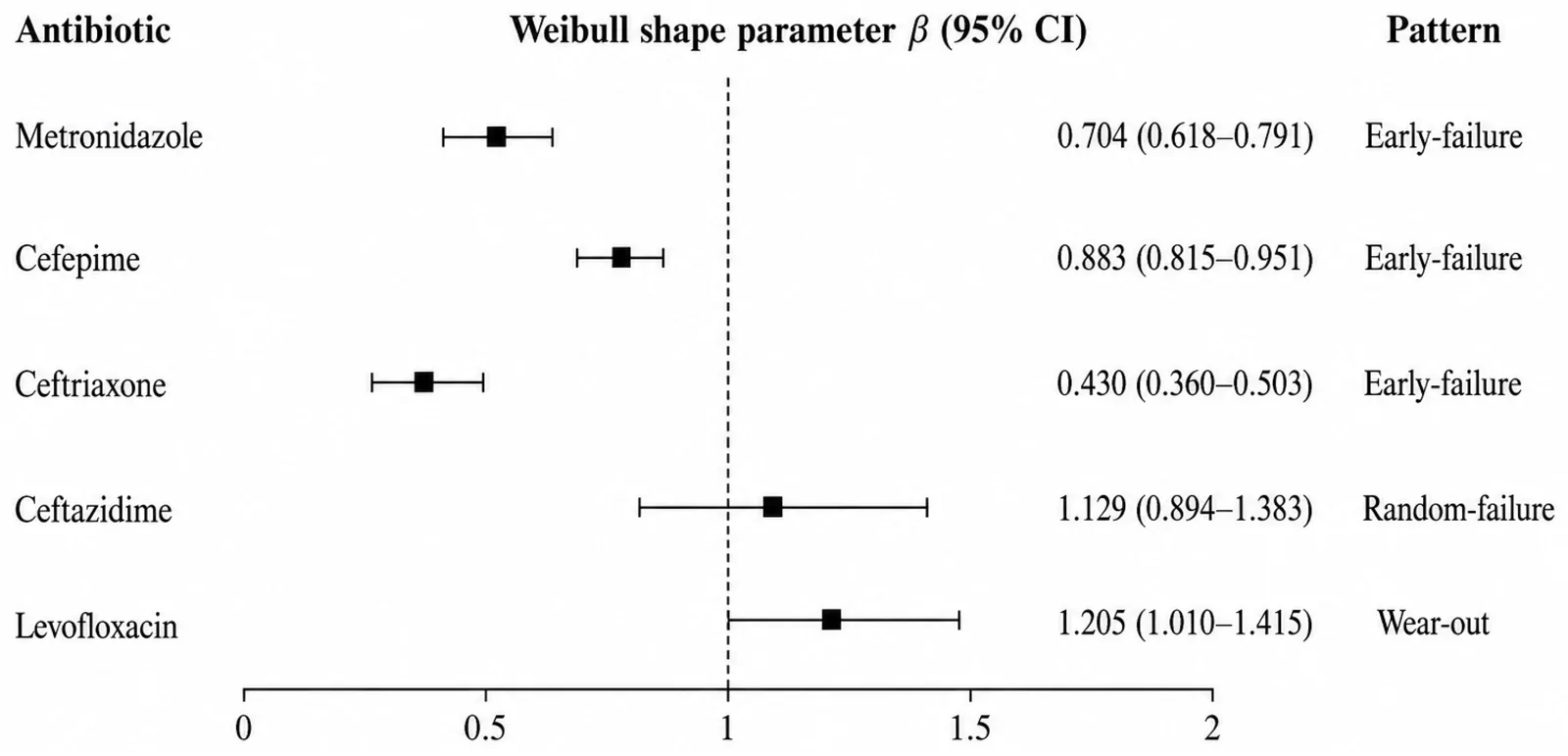
Weibull shape parameters (β) with 95% confidence intervals for time to onset of encephalopathy-related reports in FAERS. The dashed vertical line indicates β = 1. β < 1 indicates an early-failure pattern, β = 1 a random-failure pattern, and β > 1 a wear-out pattern.

The Weibull analysis showed early-failure patterns for metronidazole (β 0.704, 95% CI 0.618–0.791), cefepime (β 0.883, 95% CI 0.815–0.951), and ceftriaxone (β 0.430, 95% CI 0.360–0.503). Ceftazidime showed a random-failure pattern (β 1.129, 95% CI 0.894–1.383), whereas levofloxacin showed a modest wear-out pattern (β 1.205, 95% CI 1.010–1.415) (Figure 5).

### 3.11. Seriousness Criteria of Reports in FAERS

Seriousness criteria were frequently recorded among encephalopathy reports (Table 4). Death was recorded in 11.1% of metronidazole reports, 16.8% of cefepime reports, 14.0% of ceftriaxone reports, 11.1% of ceftazidime reports, and 12.0% of levofloxacin reports. Hospitalization was recorded in 56.6%, 66.8%, 64.9%, 50.0%, and 84.2% of reports, respectively. Life-threatening criteria were recorded in 9.4% of metronidazole reports, 20.1% of cefepime reports, 17.1% of ceftriaxone reports, 22.2% of ceftazidime reports, and 9.3% of levofloxacin reports.

## 4. Discussion

In this dual-database pharmacovigilance study, we systematically compared the reporting characteristics of encephalopathy associated with five clinically important antibiotics across the Japanese (JADER) and US (FAERS) spontaneous reporting systems. Three findings stand out. First, all five antibiotics—metronidazole, cefepime, ceftriaxone, ceftazidime, and levofloxacin—showed significant reporting disproportionality for encephalopathy in both databases, with metronidazole and cefepime showing the largest RORs in each. Because neurotoxicity is already a well-documented adverse reaction of these agents, and of cefepime and metronidazole in particular [5,7,8], these findings represent the confirmation of established adverse drug reactions within spontaneous reporting systems rather than the detection of new safety signals [11]. Second, antibiotic-associated encephalopathy was not reported as a single, uniform adverse event: the co-reported neurological manifestations, the temporal pattern of onset, the patient age distribution, and the associated renal terms differed substantially between agents. Third, in FAERS, metronidazole and cefepime—the two agents with the largest RORs—showed two clearly contrasting reported profiles, the former a delayed cerebellar syndrome and the latter an early-onset, renal-associated altered-mental-status/myoclonus syndrome, each of which closely reproduced the description of that syndrome in the clinical literature. Taken together, these observations are consistent with the concept, proposed on clinical grounds [5], that antibiotic-associated encephalopathy is best understood not as one entity but as a family of clinically and mechanistically distinct syndromes. It should be emphasized, however, that what was observed in both reporting systems was the reporting disproportionality itself; the contrasting symptom profiles were characterized in FAERS and were not reproduced when the same analysis was applied to JADER (Section 3.6).

### 4.1. Cross-Database Consistency and Interpretation of Reporting Disproportionality

All five antibiotics showed significant reporting disproportionality for encephalopathy in both JADER and FAERS, and the disproportionality persisted in the sensitivity analysis restricted to PT Encephalopathy alone. Because the term Toxic encephalopathy may be applied preferentially to events already suspected to be drug-related, its exclusion provides reassurance that the findings were not merely an artifact of terminology-driven reporting. The absolute magnitude of the ROR differed between the two systems, which is expected: the reporting odds ratio is a measure of relative reporting within a given database and is strongly influenced by the composition of the background reports, national prescribing patterns, differences in regulatory reporting culture, and notoriety or stimulated reporting for particular drug-event pairs [10]. For this reason, the ROR should not be compared as an absolute quantity across databases, and we instead emphasize that disproportionate reporting was present in both systems for all five agents. The RORs for ceftriaxone, ceftazidime, and levofloxacin were of broadly similar magnitude in the two systems, whereas metronidazole produced a markedly higher ROR in JADER and cefepime the largest ROR in FAERS. Rather than a true difference in biological risk, the disproportionately large metronidazole ROR in JADER may partly reflect heightened recognition and reporting of metronidazole-induced encephalopathy in Japan, as well as differences in the overall composition of reports between the two databases [12]. The convergence of two structurally different reporting systems on the same qualitative conclusion is a strength of the study, but it should be read as evidence of reproducible reporting patterns rather than as evidence about the incidence or comparative risk of encephalopathy.

### 4.2. Metronidazole: A Delayed Cerebellar Profile

Metronidazole showed the largest ROR in JADER (186.70) and the second largest in FAERS (29.11), and its reported clinical fingerprint was distinctive. Among encephalopathy reports, ataxia, dysarthria, and gait disturbance were markedly over-represented relative to metronidazole reports without encephalopathy (ataxia OR 12.48, dysarthria OR 11.11, gait disturbance OR 5.94), a pattern that maps directly onto a cerebellar syndrome. This cerebellar profile is concordant with the established entity of metronidazole-induced encephalopathy. Although imaging data are not available in spontaneous reporting databases, that entity is clinically defined by dysarthria, gait instability, and limb dyscoordination and, in a systematic review of 136 patients, by reversible symmetric T2/FLAIR hyperintensity of the dentate nuclei in 90% of cases [8,13]. Importantly, the association is not confined to spontaneous reports: in a population-based nested case–control study of adults aged 66 years or older, metronidazole was associated with an increased adjusted risk of neurologic events relative to clindamycin (adjusted OR 1.43; central nervous system events adjusted OR 1.46), with a cumulative-dose relationship [14], and in a recent single-center cohort of 92,838 metronidazole recipients, neurologic adverse events occurred at 0.05 per 1000 patient-days, presented predominantly with dysarthria, ataxia, and seizures, and were independently associated with liver cirrhosis, chronic kidney disease, intravenous administration, and low body weight [15]. The temporal pattern reinforced this cerebellar interpretation and its cumulative-exposure mechanism: in JADER, the median time to onset for metronidazole was 28 days (IQR 14–46), with only 13.1% of events occurring within the first week—substantially later than for the cephalosporins. The reported patient with metronidazole-associated encephalopathy therefore has a recognizable silhouette that contrasts point for point with the cefepime patient described below: a patient in late middle age (median 65 years, younger than the cefepime patients), who has been receiving metronidazole for several weeks rather than several days, and who develops progressive dysarthria, gait instability, and limb ataxia—a cerebellar syndrome in which myoclonus (1.8% of reports) and depressed consciousness are comparatively uncommon—with renal-associated terms only modestly over-represented (OR 1.53), in keeping with a cumulative-dose mechanism rather than acute accumulation due to impaired clearance. Within the widely used clinical classification of AAE, this delayed, MRI-positive cerebellar presentation corresponds to the type characteristically attributed to metronidazole and distinct from the acute encephalopathies caused by beta-lactams [5]. Our cross-database reporting data are therefore consistent with, and add reporting-system evidence to, a syndrome previously defined mainly through case series, imaging studies, and analytic cohorts; they do not define a new syndrome.

### 4.3. Cefepime and the Cephalosporins: An Early, Renal-Associated Altered-Mental-Status Profile

Cefepime displayed a sharply contrasting profile and the largest ROR in FAERS (69.29). Its encephalopathy reports were dominated by depressed level of consciousness (OR 12.88), myoclonus (OR 11.76), confusional state (OR 3.39), and seizure (OR 2.66)—an acute encephalopathy with prominent myoclonus and impaired awareness rather than cerebellar signs. This profile closely reproduces the previously described syndrome of cefepime neurotoxicity. In the systematic review by Payne et al., affected patients (median age 69 years, 80% with renal dysfunction) uniformly showed altered mental status, most commonly reduced consciousness (47%), myoclonus (42%), and confusion (42%) [7]. Mechanistically, cefepime neurotoxicity is attributed to concentration-dependent antagonism of GABA-A receptors, and its best-established risk factor is impaired renal clearance leading to drug accumulation [5,7]; a 2025 meta-analysis of 23 studies identified an estimated glomerular filtration rate below 30 mL/min/1.73 m^2^ as the dominant predisposing factor (OR approximately 10), with older age, pre-existing central nervous system disease, and inappropriate dosing as additional contributors [16]. Comparative evidence further indicates that this neurotoxicity is a property of cefepime rather than of broad-spectrum beta-lactam therapy in general: in the ACORN randomized trial of 2511 hospitalized adults, patients assigned to cefepime had fewer days alive and free of delirium and coma within 14 days than those assigned to piperacillin-tazobactam (mean 11.9 vs. 12.2 days; OR 0.79) [17]. Our reporting data reproduced this profile in each of its dimensions. The temporal signature was early: in JADER, the median onset was 4 days (IQR 3–6), with 80.8% of events occurring within one week—closely matching the median onset of 4 days reported by Payne et al. [7]. Renal-associated terms were co-reported far more often in cefepime encephalopathy reports than in cefepime reports without encephalopathy (29.7% vs. 12.8%; OR 2.88), dialysis-related terms were over-represented (OR 2.72), and decreased-GFR terms, although rare, were disproportionately reported (OR 13.17). Patients were older (median 71 years), consistent with reduced renal reserve as a susceptibility factor. Together, these findings are consistent with a coherent renal-associated, altered-mental-status/myoclonus profile—accumulation of a renally cleared cephalosporin in older or renally impaired patients producing an acute encephalopathy—corresponding to the seizure/myoclonus (type 1) category of the clinical classification [5]. The elevated renal-associated reporting seen for ceftriaxone (OR 3.18) and ceftazidime, and the older age of ceftazidime cases (median 77.5 years), are compatible with a shared beta-lactam mechanism across the cephalosporin class, with cefepime at the extreme, and with a recent hospital cohort in which reduced renal function was an independent predictor of AAE [6]. Clinically, these observations support renal-function-based dose adjustment, heightened vigilance in elderly and dialysis-dependent patients, and early consideration of drug toxicity when myoclonus and reduced consciousness develop during cefepime therapy.

### 4.4. Levofloxacin and the Laboratory-Abnormality-Related Terms

Levofloxacin showed the smallest, although still significant, reporting disproportionality for encephalopathy in both databases. Fluoroquinolone-associated central nervous system effects are recognized and are thought to involve enhanced neuronal excitability, including actions on GABA-A and N-methyl-D-aspartate receptors [18]. Levofloxacin-associated neurological events have been described predominantly in older patients and in those with renal impairment, in whom reduced clearance leads to drug accumulation [19,20], consistent with the elevated co-reported renal-associated terms observed here (OR 7.26). Among the exploratory laboratory-abnormality terms, hyperkalemia-related terms were disproportionately reported in levofloxacin encephalopathy cases (OR 42.03); however, this estimate rests on a small number of events and is highly susceptible to confounding by renal dysfunction, disease severity, acute infection, and concomitant medications, as well as to selective co-reporting of abnormal results. It should therefore be regarded as a hypothesis-generating marker of the metabolic milieu in which severe encephalopathy is reported—most plausibly a marker of renal and systemic illness—rather than as evidence of a causal biochemical pathway.

### 4.5. Seriousness of the Reports and Clinical Implications

Encephalopathy reports for all five agents frequently carried seriousness criteria. Hospitalization was recorded in a majority of reports, death in 11–17%, and life-threatening criteria most often for cefepime (20.1%) and ceftazidime (22.2%). The FAERS outcome fields describe the seriousness of the reported case as a whole, not the severity of the encephalopathy itself, and a report may carry the criterion death or life-threatening because of the underlying infection, organ failure, or another adverse event recorded in the same case rather than because of the encephalopathy. The higher frequency of death and life-threatening criteria among cefepime reports is therefore most plausibly explained by the population in which cefepime is used—hospitalized, often critically ill, and frequently renally impaired patients—rather than by a more severe form of encephalopathy, and no conclusion about differences in encephalopathy severity between antibiotics can be drawn from these data. What these figures do indicate is that encephalopathy attributed to antibiotics is reported predominantly in seriously ill patients, in whom it may be difficult to distinguish from sepsis-associated, metabolic, or other encephalopathies. Because antibiotic-associated encephalopathy is frequently reversible once the offending drug is discontinued—particularly with metronidazole and cefepime [7,8]—clinicians should maintain a low threshold for considering an antibiotic cause when new neurological deterioration develops during therapy, bearing in mind that the expected presentation is agent-specific and that the diagnosis remains one of exclusion in this setting.

### 4.6. Systematic Comparison with Previous Evidence

Table 5 summarizes, for each antibiotic, the principal previous evidence on encephalopathy or neurotoxicity and the corresponding findings of the present study, together with an assessment of concordance. Two points emerge. First, all five antibiotics showed reporting disproportionality in both databases, with metronidazole and cefepime showing the largest RORs in each; the detailed clinical profile—symptom pattern (cerebellar versus myoclonus/impaired consciousness), patient age, and renal association—was characterized in FAERS, where the necessary fields are populated [7,8,14,15,16,17]. When the co-reported symptom analysis was repeated in JADER, this symptom-level contrast was not reproduced (Supplementary Table S5; Section 3.6), possibly because of a difference in coding practice, although a true between-population difference cannot be excluded. The present study therefore demonstrates, in two independent reporting systems, the reproducibility of the reporting disproportionality, and confirms in FAERS a detailed clinical picture consistent with syndromes previously characterized in case series, systematic reviews, an analytic case–control study, a hospital cohort, and a randomized trial. Second, where the previous evidence is thinner—ceftriaxone, ceftazidime, and levofloxacin—the reporting data are consistent with the available case-based literature (early onset, older age, and renal association for the cephalosporins; renal association for levofloxacin [19,20]) but do not permit a detailed profile, mainly because of smaller numbers of reports. Compared with the recent FAERS-based screen of 121 antibiotics [9], which assigned drugs to the three clinical types on the basis of prior literature and used an 87-term outcome definition, the present study examined the reported manifestations directly and in two reporting systems, using a narrower two-term outcome definition; the higher RORs observed here reflect that narrower definition rather than discordant findings, and the two approaches should be read as complementary. One apparent discordance deserves comment. For metronidazole, the FAERS report-date analysis yielded a short median onset (8 days here and approximately 5 days in the recent FAERS-based screen [9]), which is at odds with the delayed, cumulative-exposure course expected of metronidazole cerebellar toxicity; the event-specific onset dates available in JADER recovered the expected delayed course (median 28 days), in agreement with the analytic and systematic-review evidence [8,14]. This discordance may be explained by the report-level nature of the FAERS event date rather than by a true difference between populations, and it illustrates both the value of examining more than one reporting system and the limits of FAERS timing data.

### 4.7. Strengths and Limitations

The principal strengths of this study are its dual-database design, which allows the reproducibility of reporting patterns to be tested across two independent populations and reporting cultures; the rigorous FAERS deduplication yielding a well-defined analytic population of 20,435,080 unique cases; the pre-specified sensitivity analysis addressing terminology bias; and the multidimensional characterization of each drug’s encephalopathy beyond a single disproportionality metric, in accordance with contemporary READUS-PV reporting recommendations. Because reporting disproportionality was present in two independent reporting systems rather than resting on a single database, with metronidazole and cefepime showing the largest RORs in each, these findings are less likely to be database-specific artifacts; the detailed clinical profiles, by contrast, were characterized mainly in FAERS, and the symptom-level contrast was not reproduced when the same analysis was applied to JADER (Section 3.6). Reproduction across two systems does not, however, protect against biases that both systems share, such as label-driven reporting and confounding by the populations in which each antibiotic is used.

Several limitations, inherent to spontaneous reporting systems, must be emphasized. First, these data cannot establish causation, incidence, or relative risk. Spontaneous reports lack a denominator of exposed patients, are subject to substantial under-reporting, and are affected by reporting biases including notoriety and stimulated reporting. The ROR quantifies disproportionate reporting only, and the ROR of each antibiotic is shaped by its prescribing frequency, indications, treatment duration, healthcare setting, and reporting practices. Levofloxacin and ceftriaxone, for example, are prescribed far more widely than cefepime, largely in outpatient or general-ward settings, so that encephalopathy reports are diluted within a very large background of other reports; cefepime is used predominantly in hospitalized and critically ill patients, in whom encephalopathy is both more likely to occur and more likely to be recognized and reported. A larger ROR for metronidazole or cefepime therefore does not imply that these agents are more neurotoxic, or that they carry a higher clinical risk of encephalopathy, than ceftriaxone or levofloxacin. Second, and most importantly, confounding by underlying comorbidity and disease severity cannot be controlled and may account for part of the associations observed. Encephalopathy is common among hospitalized and critically ill patients and has many causes—sepsis-associated encephalopathy, uremic and hepatic encephalopathy, electrolyte disturbance, hypoxia, and sedative or opioid medication among them—and the antibiotics studied here are given precisely to patients at risk of these conditions. Cefepime in particular is used in intensive care units and in febrile neutropenia, and metronidazole is used in patients with liver disease, in whom hepatic encephalopathy may be misattributed to the drug. The renal and laboratory associations are especially vulnerable to this confounding: renal dysfunction is both a plausible causal factor (reduced drug clearance) and a marker of the severe illness in which encephalopathy of any cause is more likely, and these data cannot separate the two. The renal-associated and laboratory terms were co-reported adverse-event terms, not systematically ascertained baseline comorbidities or measured laboratory values, and their absence in a report does not indicate true absence. Third, the reported symptom patterns may be shaped by reporting bias arising from clinician awareness and product labeling. Cerebellar signs such as ataxia and dysarthria are described in the product information and textbook descriptions of metronidazole neurotoxicity, and myoclonus, seizures, and encephalopathy are described for cefepime, which has also been the subject of a regulatory safety communication [21]. A clinician who recognizes a reaction after consulting the product information is more likely to record it and to describe it in the terms used there, so that the observed profiles may partly reflect the existing descriptions rather than independently observed clinical features; the reproduction of these profiles across two databases does not exclude this circularity, because both are subject to the same labeling. Fourth, the study did not formally identify phenotypes. The agent-specific profiles were obtained by comparing predefined co-reported manifestations, patient characteristics, and timing between antibiotics, not by unsupervised clustering or latent-class analysis, and the term profile is used in this sense throughout. The neurological manifestations were co-reported terms and do not establish a reliable chronological sequence relative to the encephalopathy diagnosis; they should be read as the reported clinical picture rather than as verified presenting symptoms. Fifth, the seriousness criteria (death, hospitalization, life-threatening) describe the reported case, not the encephalopathy, and cannot support conclusions about differences in encephalopathy severity between antibiotics. Sixth, the FAERS time-to-onset and Weibull analyses are exploratory because EVENT_DT is a report-level date that is not specific to the encephalopathy PT; the corresponding JADER onset dates, though event-specific, were available for limited numbers of cases, and the ceftazidime estimates in particular rest on very few evaluable reports. Seventh, the exploratory renal, laboratory, and individual-manifestation analyses involved multiple comparisons and were interpreted according to effect size, precision, biological plausibility, and consistency rather than statistical significance alone; the small-cell odds ratios should be viewed as hypothesis-generating. Finally, coding practices, drug-name harmonization, and the availability of demographic and date fields differ between JADER and FAERS, which contributes to the differing absolute magnitudes of the RORs and warrants the cautious, qualitative interpretation adopted here.

## 5. Conclusions

Using two independent spontaneous reporting systems, we confirmed that reporting disproportionality for encephalopathy is present for five antibiotics in both databases, with metronidazole and cefepime showing the largest RORs in each—consistent with adverse reactions that are already well documented—and showed that the co-reported clinical features, timing, patient age, and renal-associated terms of these encephalopathies differ systematically between agents. The contrast between a delayed cerebellar profile for metronidazole and an early, renal-associated altered-mental-status/myoclonus profile for cefepime was characterized mainly in the US (FAERS) data—and is consistent with syndromes previously described in case series, systematic reviews, and clinical studies and with the established clinical classification of antibiotic-associated encephalopathy—whereas the symptom-level contrast was not reproduced in the Japanese data, a discrepancy that may reflect differences in coding practice. These findings indicate that antibiotic-associated encephalopathy is reported with agent-specific profiles rather than as a single uniform event. Awareness of these patterns may facilitate earlier recognition, prompt drug discontinuation, and renal-function-based dose adjustment; confirmation in analytic studies with exposure denominators and adjustment for confounding is warranted.

## Figures and Tables

**Table 1:** Disproportionality analysis of antibiotic-associated encephalopathy in JADER and FAERS.

Antibiotic	JADER Suspected-Drug Reports, *n*	JADER Encephalopathy Reports, *n*	JADER ROR (95% CI)	FAERS PS/SS Reports, *n*	FAERS Encephalopathy Reports, *n*	FAERS ROR (95% CI)
Metronidazole	1269	586	186.70 (166.59–209.23)	30,522	933	29.11 (27.23–31.11)
Cefepime	1266	270	55.33 (48.23–63.47)	6868	488	69.29 (63.13–76.04)
Ceftriaxone	3945	184	9.79 (8.43–11.38)	25,375	228	8.11 (7.12–9.25)
Ceftazidime	469	21	9.13 (5.89–14.15)	3794	54	12.83 (9.81–16.79)
Levofloxacin	6431	82	2.53 (2.03–3.15)	50,630	183	3.24 (2.80–3.74)

CI, confidence interval; FAERS, US FDA Adverse Event Reporting System; JADER, Japanese Adverse Drug Event Report database; PS, primary suspect; ROR, reporting odds ratio; SS, secondary suspect. Encephalopathy was defined using the MedDRA Preferred Terms Encephalopathy and Toxic encephalopathy. In JADER, exposure was defined as a suspected drug; in FAERS, exposure was defined as a primary or secondary suspect drug. RORs indicate reporting disproportionality and should not be interpreted as incidence, relative risk, or causal effect.

**Table 2:** Time to onset of encephalopathy in JADER.

Antibiotic	Evaluable Cases, *n*	Median (IQR), Days	≤7 Days, %	≤14 Days, %	≤30 Days, %
Metronidazole	145	28 (14–46)	13.1	29.0	57.9
Cefepime	78	4 (3–6)	80.8	94.9	98.7
Ceftriaxone	38	5.5 (4.25–7)	84.2	97.4	97.4
Ceftazidime	4	3.5 (2–6.25)	75.0	100	100

IQR, interquartile range. Time to onset was calculated from the treatment start date to the encephalopathy onset date; only cases with valid dates were included.

**Table 3:** Time to onset of encephalopathy-related reports in FAERS.

Antibiotic	Evaluable Cases, *n*	Median (IQR), Days	≤7 Days, %	≤14 Days, %	≤30 Days, %
Metronidazole	109	8 (4–21)	47.7	58.7	83.5
Cefepime	265	6 (4–14)	54.0	77.7	96.6
Ceftriaxone	61	6 (1–12)	59.0	83.6	93.4
Ceftazidime	42	6 (4.25–12)	52.4	78.6	95.2
Levofloxacin	77	6 (4–13)	54.5	75.3	97.4

IQR, interquartile range. TTO was calculated from THER START_DT to DEMO EVENT_DT using exact calendar dates. Because EVENT_DT is a report-level date and is not necessarily specific to the encephalopathy Preferred Term, these results should be interpreted as exploratory.

**Table 4:** Seriousness criteria recorded among FAERS encephalopathy reports.

Antibiotic	Encephalopathy Cases (*n*)	Death, *n* (%)	Hospitalization, *n* (%)	Life-Threatening, *n* (%)
Metronidazole	933	104 (11.1)	528 (56.6)	88 (9.4)
Cefepime	488	82 (16.8)	326 (66.8)	98 (20.1)
Ceftriaxone	228	32 (14.0)	148 (64.9)	39 (17.1)
Ceftazidime	54	6 (11.1)	27 (50.0)	12 (22.2)
Levofloxacin	183	22 (12.0)	154 (84.2)	17 (9.3)

Outcome categories were not mutually exclusive because more than one criterion could be recorded for the same report. Seriousness criteria refer to the report as a whole and do not indicate the severity of the encephalopathy.

**Table 5:** Systematic comparison of the present findings with previous evidence on antibiotic-associated encephalopathy and neurotoxicity.

Antibiotic	Previous Evidence (Source; Key Features)	Present Study (JADER/FAERS)	Concordance and Comment
Metronidazole	Systematic review of 136 cases [8]: cerebellar dysfunction (dysarthria, ataxia), dentate-nucleus lesions in 90%, onset after weeks of therapy. Nested case–control study [14]: adjusted OR 1.43 vs. clindamycin, cumulative-dose relationship. Hospital cohort [15]: 0.05 events per 1000 patient-days; dysarthria, ataxia, seizures; risk factors: cirrhosis, CKD, intravenous route, low body weight. Clinical classification [5]: delayed cerebellar type.	ROR 186.70 (JADER), 29.11 (FAERS). Ataxia OR 12.48, dysarthria OR 11.11, gait disturbance OR 5.94; myoclonus 1.8%. Median TTO 28 days (JADER; 13.1% within 7 days); 8 days (FAERS, report-level date). Median age 65 years. Renal-associated terms OR 1.53.	Concordant for symptom pattern (FAERS), delayed onset (JADER), and weak renal association. FAERS TTO shorter than expected, possibly attributable to the report-level event date. No new features identified.
Cefepime	Systematic review [7]: median age 69 years, 80% renal dysfunction; reduced consciousness 47%, myoclonus 42%, confusion 42%; median onset 4 days. Meta-analysis of 23 studies [16]: eGFR <30 mL/min/1.73 m2 the dominant risk factor (OR approximately 10); age, CNS disease, inappropriate dosing. ACORN trial [17]: fewer delirium- and coma-free days vs. piperacillin-tazobactam (11.9 vs. 12.2 days; OR 0.79). Hospital cohort [6]: reduced renal function an independent predictor of AAE. Classification [5]: early seizure/myoclonus type.	ROR 55.33 (JADER), 69.29 (FAERS). Depressed consciousness OR 12.88, myoclonus OR 11.76, confusional state OR 3.39, seizure OR 2.66. Median TTO 4 days (JADER; 80.8% within 7 days); 6 days (FAERS). Median age 71 years. Renal-associated terms: 29.7% vs. 12.8% (OR 2.88); dialysis OR 2.72.	Concordant for symptom pattern (FAERS), early onset, older age, and renal association. Confirms an established reaction; renal association cannot distinguish causal accumulation from confounding by illness severity.
Ceftriaxone	Case reports and series of encephalopathy, myoclonus, and non-convulsive status epilepticus, predominantly in older patients and in renal impairment; classification [5]: early seizure/myoclonus type (beta-lactam).	ROR 9.79 (JADER), 8.11 (FAERS). Median TTO 5.5 days (JADER; 84.2% within 7 days). Median age 68 years. Renal-associated terms OR 3.18.	Concordant for early onset and renal association. Symptom profile not characterized in detail because of fewer reports.
Ceftazidime	Case reports of encephalopathy, myoclonus, and seizures, mainly in elderly patients with renal failure or on dialysis; classification [5]: early seizure/myoclonus type.	ROR 9.13 (JADER), 12.83 (FAERS). Median TTO 3.5 days (JADER; n = 4). Median age 77.5 years. Renal-associated terms OR 1.43 (95% CI 0.61–3.38).	Concordant for early onset and older age; renal association imprecise because of small numbers. Estimates should be regarded as exploratory.
Levofloxacin	Reviews of fluoroquinolone neurotoxicity [18]; case reports of delirium and neurological events in renal dysfunction and elderly patients [19,20]; classification [5]: early psychiatric type.	ROR 2.53 (JADER), 3.24 (FAERS). Median TTO 6 days (FAERS). Median age 63 years. Renal-associated terms OR 7.26; hyperkalemia-related terms OR 42.03 (small numbers).	Concordant for renal association. Smallest ROR, consistent with very high prescribing volume and predominantly psychiatric rather than encephalopathy coding; not evidence of low risk. Laboratory associations hypothesis-generating.

AAE, antibiotic-associated encephalopathy; CI, confidence interval; CKD, chronic kidney disease; CNS, central nervous system; eGFR, estimated glomerular filtration rate; FAERS, US FDA Adverse Event Reporting System; JADER, Japanese Adverse Drug Event Report database; OR, odds ratio; ROR, reporting odds ratio; TTO, time to onset. ORs for manifestations and renal-associated terms compare encephalopathy with non-encephalopathy reports within each antibiotic in FAERS. RORs indicate disproportionate reporting and cannot be compared between antibiotics as measures of relative risk.

## Data Availability

The datasets analyzed in this study are publicly available. JADER can be obtained from the Pharmaceuticals and Medical Devices Agency (PMDA) of Japan, and FAERS quarterly data files from the US Food and Drug Administration (FDA).

## Supplementary Materials

The following supporting information can be downloaded at: https://www.mdpi.com/article/10.3390/antibiotics15090917/s1, Table S1: MedDRA terms used to define the outcome and co-reported covariates; Table S2: Two-by-two contingency counts for encephalopathy in FAERS; Table S3: Encephalopathy reports and reporting odds ratios by antibiotic in JADER; Table S4: Sensitivity analysis using the Preferred Term Encephalopathy alone in JADER; Table S5: Co-reported neurological manifestations in JADER encephalopathy reports for metronidazole and cefepime.
