# Supplementary material for: Agent-Specific Clinical Profiles of Antibiotic-Associated Encephalopathy: A Dual-Database Pharmacovigilance Study Using JADER and FAERS
Source: Antibiotics (Basel). 2026 Sep 17;15(9):917. doi: 10.3390/antibiotics15090917 (PMC13603405; doi:10.3390/antibiotics15090917)
Supplement: Supplementary file 1 [file antibiotics-15-00917-s001.zip › antibiotics-4552015-supplementary.pdf]

Supplementary Materials

Agent-Specific Clinical Profiles of Antibiotic-Associated Encephalopathy: A Dual-Database Pharmacovigilance Study Using JADER and FAERS

Hideya Itagaki

Supplementary Methods

Adverse events and co-reported clinical conditions were identified using Medical Dictionary for Regulatory Activities (MedDRA) terminology. The analysis was conducted using Preferred Terms (PTs) corresponding to the concepts listed in Table S1. The primary outcome was defined as reports containing either the PT Encephalopathy or Toxic encephalopathy. A sensitivity analysis was performed using Encephalopathy alone.

For categories comprising multiple terms, including renal-associated conditions, laboratory abnormalities, and neurological manifestations, a report was considered positive if at least one constituent term was reported.

Reports were classified according to the five selected active ingredients: metronidazole, cefepime, ceftriaxone, ceftazidime, and levofloxacin. Antibiotic exposure was identified among drugs reported as primary or secondary suspects in FAERS and as suspected drugs in JADER.

Because the analyses used publicly available spontaneous reporting data spanning multiple years, reports may have been coded using different versions of MedDRA across the study period. The MedDRA version was therefore not treated as a uniform analytical variable.

Table S1. MedDRA terms used to define the outcome and co-reported covariates.

Category	Terms
Primary outcome (encephalopathy)	Encephalopathy; Toxic encephalopathy
Sensitivity analysis	Encephalopathy
Renal-associated terms	Renal impairment; Chronic kidney disease; Acute kidney injury; Renal failure; Dialysis; Haemodialysis
Dialysis-related terms (subset)	Dialysis; Haemodialysis
Laboratory-abnormality-related terms	Blood creatinine increased; Glomerular filtration rate decreased; Blood urea increased; Hyponatraemia; Hypernatraemia; Hypokalaemia; Hyperkalaemia; Hypoglycaemia; Hyperglycaemia; Hepatic enzyme increased; Alanine aminotransferase increased; Aspartate aminotransferase increased; Blood bilirubin increased
Neurological manifestations	Ataxia; Dysarthria; Gait disturbance; Myoclonus; Confusional state; Depressed level of consciousness; Delirium; Seizure

Abbreviation: PT, Preferred Term. Terms are grouped according to the clinical concepts evaluated in the analysis. For categories comprising multiple terms, a report was considered positive if at least one constituent term was present.

Table S2. Two-by-two contingency counts for encephalopathy in FAERS.

Antibiotic	a: Drug + encephalopathy	b: Drug, no encephalopathy	c: Other drugs + encephalopathy	d: Other drugs, no encephalopathy	ROR (95% CI)
Metronidazole	933	29,589	22,082	20,382,476	29.11 (27.23–31.11)

Cefepime	488	6,380	22,527	20,405,685	69.29 (63.13–76.04)
Ceftriaxone	228	25,147	22,787	20,386,918	8.11 (7.12–9.25)
Ceftazidime	54	3,740	22,961	20,408,325	12.83 (9.81–16.79)
Levofloxacin	183	50,447	22,832	20,361,618	3.24 (2.80–3.74)

The FAERS analytic population comprised 20,435,080 unique reports, including 23,015 reports containing Encephalopathy or Toxic encephalopathy. Exposure was defined as the antibiotic being reported as a primary or secondary suspect drug. The reporting odds ratio (ROR) was calculated as (a x d)/(b x c).

Table S3. Encephalopathy reports and reporting odds ratios by antibiotic in JADER.

Antibiotic	Drug + encephalopathy	Drug, no encephalopathy	ROR (95% CI)
Metronidazole	586	683	186.70 (166.59–209.23)
Cefepime	270	996	55.33 (48.23–63.47)
Ceftriaxone	184	3,761	9.79 (8.43–11.38)
Ceftazidime	21	448	9.13 (5.89–14.15)
Levofloxacin	82	6,349	2.53 (2.03–3.15)

Exposure was defined as the antibiotic being reported as a suspected drug.

Table S4. Sensitivity analysis using the Preferred Term Encephalopathy alone in JADER.

Antibiotic	ROR (95% CI)
Metronidazole	32.59 (26.43–40.19)
Cefepime	20.82 (16.18–26.80)
Ceftazidime	4.88 (2.18–10.94)
Ceftriaxone	4.60 (3.44–6.14)
Levofloxacin	1.71 (1.19–2.47)

Reporting signals remained statistically significant for all five antibiotics when the outcome definition was restricted to the PT encephalopathy, and the PT Toxic encephalopathy was excluded.

Table S5. Co-reported neurological manifestations in JADER encephalopathy reports for metronidazole and cefepime.

Manifestation	Metronidazole, n (%)	Metronidazole OR (95% CI)	Cefepime, n (%)	Cefepime OR (95% CI)
Ataxia	4 (0.7)	2.34 (0.43–12.82)	0 (0)	NE
Dysarthria	5 (0.9)	0.97 (0.29–3.20)	1 (0.4)	3.70 (0.23–59.33)
Gait disturbance	5 (0.9)	1.46 (0.39–5.47)	0 (0)	NE
Myoclonus	0 (0)	NE	0 (0)	NE
Depressed level of consciousness	4 (0.7)	0.78 (0.22–2.76)	5 (1.9)	1.23 (0.44–3.43)
Confusional state	0 (0)	NE	0 (0)	NE
Delirium	0 (0)	NE	2 (0.7)	0.49 (0.11–2.15)
Seizure	3 (0.5)	0.12 (0.04–0.38)	1 (0.4)	0.12 (0.02–0.91)

Analysis restricted to reports in which the antibiotic was reported as a suspected drug. n (%) denotes the number and within-group percentage of encephalopathy reports recording each manifestation (metronidazole, N = 586; cefepime, N = 270). Odds ratios compare encephalopathy with non-encephalopathy reports of the same antibiotic (Wald 95% CI). NE, not estimable (zero cell). In contrast to FAERS, the prespecified co-reported neurological Preferred Terms were uncommon in JADER (all <2%), and the FAERS symptom-level contrast was not reproduced; one possible explanation is a difference in coding practice, although a true between-population difference cannot be excluded. CI, confidence interval; OR, odds ratio.
